# Cymoxanil disrupts RNA synthesis through inhibiting the activity of dihydrofolate reductase

**DOI:** 10.1038/s41598-024-62563-5

**Published:** 2024-05-22

**Authors:** Thomas David Daniel Kazmirchuk, Daniel. J. Burnside, Jiashu Wang, Sasi Kumar Jagadeesan, Mustafa Al-gafari, Eshan Silva, Taylor Potter, Calvin Bradbury-Jost, Nishka Beersing Ramessur, Brittany Ellis, Sarah Takallou, Maryam Hajikarimlou, Houman Moteshareie, Kamaleldin B. Said, Bahram Samanfar, Eugene Fletcher, Ashkan Golshani

**Affiliations:** 1 Department of Biology and the Ottawa Institute of Systems Biology (OISB), Carleton University, Ottawa, K1S 5B6 Canada; 2 Department of Pathology and Microbiology, University of Hail, 55476 Hail, Saudi Arabia; 3 Agriculture and Agri-Food Canada, Ottawa, K1A 0C6 Canada

**Keywords:** Chemical biology, Chemical genetics, Enzyme mechanisms, Mechanism of action

## Abstract

The agricultural fungicide cymoxanil (CMX) is commonly used in the treatment of plant pathogens, such as *Phytophthora infestans*. Although the use of CMX is widespread throughout the agricultural industry and internationally, the exact mechanism of action behind this fungicide remains unclear. Therefore, we sought to elucidate the biocidal mechanism underlying CMX. This was accomplished by first performing a large-scale chemical-genomic screen comprising the 4000 haploid non-essential gene deletion array of the yeast *Saccharomyces cerevisiae*. We found that gene families related to de novo purine biosynthesis and ribonucleoside synthesis were enriched in the presence of CMX. These results were confirmed through additional spot-test and colony counting assays. We next examined whether CMX affects RNA biosynthesis. Using qRT-PCR and expression assays, we found that CMX appears to target RNA biosynthesis possibly through the yeast dihydrofolate reductase (DHFR) enzyme Dfr1. To determine whether DHFR is a target of CMX, we performed an in-silico molecular docking assay between CMX and yeast, human, and *P. infestans* DHFR. The results suggest that CMX directly interacts with the active site of all tested forms of DHFR using conserved residues. Using an in vitro DHFR activity assay we observed that CMX inhibits DHFR activity in a dose-dependent relationship.

## Introduction

Plant pathogens are an ever-increasing concern for crop security and yield. To this point, crop security and destruction have been recently cited as major geopolitical and humanitarian concerns by the Food and Agriculture Organization (FAO) of the United Nations^1^. To combat destructive plant pathogens, the agricultural industry commonly employs biocidal compounds including pesticides (herbicides, insecticides, etc.) and antimicrobials (fungicides, antibiotics, antiprotozoals, etc.)^2^. While use of these biocides directly increases crop yield and security^3^, the bioactivity for many of these compounds remain unknown. One such compound is the fungicide cymoxanil (CMX; 1-(2-cyano-2-methoxyiminoacetyl)-3-ethylurea). This synthetic acetamide is currently used as foliar-applied fungicide for treatment of the causal agent of potato blight *Phytophthora infestans*, to which it displays specificity^4,5^. CMX is additionally used as a treatment for tomato blight and grape mildew^6,7^.

Little work has been invested into investigating the mechanism behind CMX, with many studies rather focused on assessing the impact of the fungicide on *P. infestans* infected crops. In a study performed by Ziogas and Davidse, CMX was found to broadly target DNA and RNA biosynthesis^8^. The authors did note that this was likely a secondary effect - an observation later supported by Andrieu and colleagues^8,9^. In terms of human toxicity, CMX is thought to be rapidly absorbed in the blood and plasma with maximum concentrations reached within four hours of dosage^10^. A 2021, CMX is reported to meet the current health and environmental safety standards of Canada and Europe nor is considered to be carcinogenic - a conclusion supported by a recent study pertaining to Iranian fruit crop consumption^11–13^. In south Asia, grapes or raisins contaminated with CMX were found to pose no risk for human consumption^14,15^. Despite its prevalent international use, details underlying the mechanism of action of CMX remain ambiguous.

**Figure 1:**
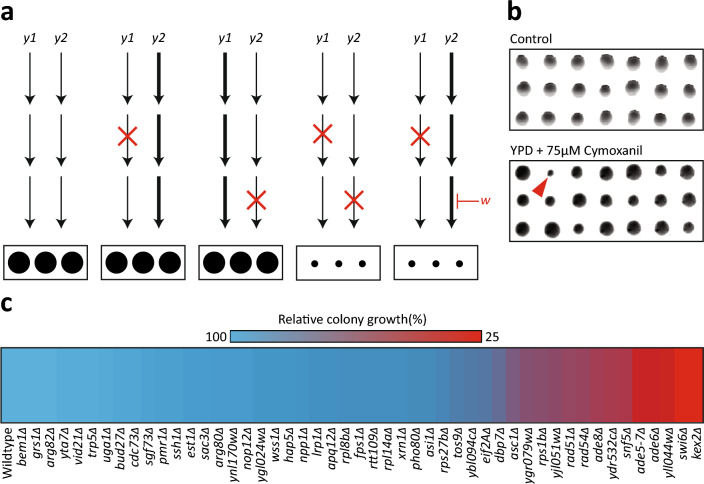
Chemical-genomic screening reveals 49 gene candidates involved in the response to CMX. (**a**) A schematic depicting the theory underlying chemical genomic screening. From left to right: two parallel genetic pathways (y1 and y2) produce a growth phenotype. When either pathway is mutated, the parallel pathway compensates. When both pathways contain mutations, no compensation can occur resulting in a decrease in growth phenotype. Coupling a single mutation with an inhibitory compound (w) mimics the effect of two mutations, resulting in a decreased growth phenotype. (**b**) Representative images from large-scale chemical genomic screening. The red arrow indicates a sensitive colony. (**c**) The sensitivities of each gene candidate to CMX derived from follow up colony size sensitivity analysis.

A common approach to study the mechanism of action for a compound is the use of chemical-genomic screening. This type of screening is based on the notion that the presence of parallel compensatory pathways can compensate for the genetic inactivation of a single pathway, thereby resulting in no obvious phenotypic consequence^16,17^. Inactivation of the second functionally overlapping pathway using a single-gene deletion mutation^18^ or a chemical inactivation^19^ can cause an unexpected phenotypic effect that can be measured. A common approach in such screens is to challenge a library of non-essential gene deletion mutants in *Escherichia coli*^20^ or *Saccharomyces cerevisiae*^21,22^ to a sub-inhibitory concentration of the target bioactive compound. Specifically, single-gene deletion libraries are challenged by the compound of interest with the resulting biological responses providing indirect evidence of genetic targets (Fig. 1a)^23–25^. This approach has been used to elucidate the molecular activity of natural bioactive compounds^26,27^, pharmaceuticals^28^, nanoparticles^29^ and herbal extracts^30^.

Thus, to investigate the mechanism of action of CMX, we employed a sensitivity screen using roughly 4000 haploid non-essential gene deletion strains of the yeast *S. cerevisiae* against CMX. Using a combination of yeast chemical genomics, molecular genetics, in-silico docking simulations, and protein activity assays, our observations suggest that CMX may disrupt RNA biosynthesis through targeting the dihydrofolate reductase (DHFR) enzyme. DHFR is a folate reductase protein that functions to convert dihydrofolate (DHF) to tetrahydrofolate (THF). The resulting THF acts as a carbon donor to key metabolites including purines. Thus, by preventing the reducing activity of DHFR, CMX indirectly inhibits purine biosynthesis through preventing the conversion of DHF to THF. The results presented in this study therefore contribute to the overall physiological and biochemical understanding of CMX.

## Results

### Chemical genomic profiling of CMX

To investigate the mechanism of action of CMX, we first determined a suitable sub-inhibitory concentration of the fungicide on the growth of *S. cerevisiae*. We subjected a random collection of 384 single-gene deletions to several concentrations of CMX (0, 12.5, 25, 37.5, 75, 150 *µ*M). The results indicate that for the investigated yeast non-essential gene deletions, 75 *µ*M of CMX results in an approximately 5% of the colonies showing a noticeable reduction in their relative growth. This frequency of sensitive mutants can represent a meaningful balance for investigating the mode of activity of a compound^19,31^. We next subjected the entire yeast single-gene deletion set to YPD media with or without 75 *µ*M CMX (Fig. 1b – representative image). After incubating the yeasts at 30 °C for 48 hours, we found that 49 yeast colonies displayed a relative growth defect of 25% or greater. These candidates were then transferred onto a manageable single plate and subjected to follow up studies that included the repeat of the sensitivity screen. Figure 1c represents a heat map corresponding to the average colony size for the 49 strains obtained from the large-scale screen.

**Figure 2:**
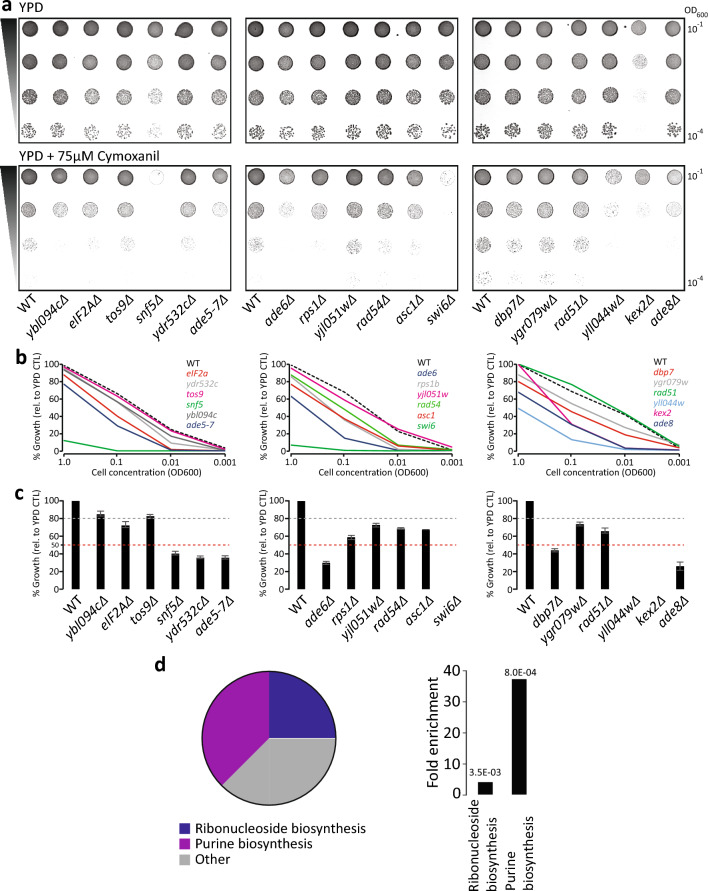
Small-scale verification of 18 gene candidates to CMX. (**a**) Spot-test analysis of the 18 candidates in the presence and absence of 75* µM* CMX. (**b**) Quantification of spot-tests in (**a**). (**c**) Colony-count analysis of the 18 candidates. Grey bar represents a 20% reduction in colony growth. The red bar represents a 50% reduction in colony growth. (**d**) GO-analysis of the 8 most-sensitive gene candidates reveals two statistically significant gene families: de novo purine biosynthesis and ribonucleoside biosynthesis.

To confirm the sensitivity of the obtained strains we next subjected these strains to spot-test analysis. Spot-test analysis offers a refined screening approach to study strain sensitivity which is complementary to the colony size approach^32^. A serial dilution of each candidate yeast (in addition to a WT) was spotted on plates with or without 75 *µ*M CMX (Fig. 2a). To quantify colony sensitivity, each spot was measured with a 4x4 region of interest for grey value intensity (Fig. 2b). This analysis resulted in 13 candidates displaying sensitivity to CMX relative to the WT control.

In parallel, we performed a colony-counting assay. We diluted the candidate yeasts from overnight culture to 1.0E–03 OD and transferred them onto plates in the presence or absence of 75 *µ*M CMX. We observed that many of the yeasts displayed a growth sensitivity relative to the WT control and appear to either be partially or extremely sensitive (Fig. 2c). We observed that 8 yeast deletion strains showed at least 50% reduction in growth (Fig. 2c). Table 1 shows the list of the gene deletion strains that were sensitive in all investigated assays. In the GO term enrichment analysis of the affected genes, *de novo* purine nucleotide biosynthesis and ribonucleoside synthesis appeared to be significantly enriched (Fig. 2d).

**Table 1:** Candidate functional descriptions derived from chemical-genomic screening in the presence of CMX.

Gene name	Gene function
*ADE5-7*	Catalyzes a step in de novo purine biosynthesis pathway
*ADE6*	Catalyzes a step in de novo purine biosynthesis pathway
*ADE58*	Catalyzes a step in de novo purine biosynthesis pathway
*SWI6*	Transcription regulation; forms complex with Swi4 and Mbp1
*SNF5*	Transcription regulation; forms complex with Snf2 and Snf6
*DBP7*	RNA processing
*KEX2*	Calcium-dependent serine protease
*YLL044w*	Dubious ORF

Due to the nature of chemical-genomic screening, the enriched families likely display an indirect effect of CMX. As purine biosynthesis is heavily enriched, the primary target of CMX is likely a protein involved in this process. *DFR1* is an essential gene in yeast that encodes for an evolutionary conserved DHFR, a key enzyme in purine biosynthesis. Being an essential gene, it is absent from the non-essential gene knockout library used here. Interestingly it is co-expressed with four of the target genes identified in our screen, namely *ADE5,7, ADE6, ADE8* and *DBP7*^33^. Also, it genetically interacts with *ADE6* and is predicted to functionally interact with *YLL044W*^33^. Consequently, we further studied nucleotide biosynthesis and Dfr1 as a potential target for CMX.

### CMX attenuates RNA biosynthesis

**Figure 3:**
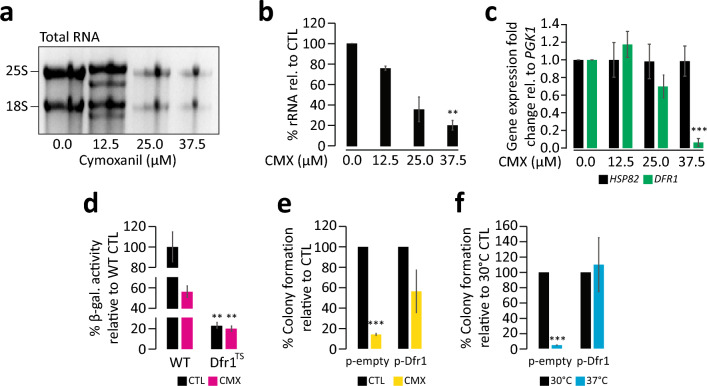
The yeast DHFR enzyme Dfr1 is a target of CMX. (**a**) total RNA was extracted from yeasts subjected to several concentrations of CMX, showing a dose-dependent reduction in rRNA band intensity obtained from a total RNA extraction, quantified in (**b**). (**c**) qRT-PCR analysis of *DRF1* and *HSC82* from RNA extracted from yeasts grown in the presence of increasing concentrations of CMX. (**d**) *β* -gal activity of WT and a temperature-sensitive knockdown of Dfr1 in the presence and absence of CMX. (**e**) Colony-counting analysis of yeasts expressing *DFR1* on a plasmid in the presence or absence of CMX. An empty vector not containing Drf1 was used as a control under the same conditions. (**f**) Using the same strains in (**e**), both were subject to 30 °C and 37 °C as a control. **Denotes significance values less than 0.01. ***Denotes significance values less than 0.001. The original gel seen in “A” can be found in Supplemental Fig. 1.

To study the effect of CMX on RNA synthesis, total RNA was extracted from yeast cells treated with different concentrations of CMX. Indicated in Fig. 3a, we observed a dose-response correlation between CMX concentration and the reduction in rRNA content obtained from total RNA extraction. As the concentration of CMX increases, we observed a corresponding decrease in rRNA band intensity (Fig. 3a). This is in accord with an activity for CMX to target RNA biosynthesis. Compared to the control (no CMX), rRNA content was reduced by approximately 80% at 37.5 *µ*M CMX (Fig. 3b).

We then assessed the expression of *DFR1* and *HSC82* (used as a control) in the presence of CMX (Fig. 3c). The *DFR1* gene encodes the yeast DHFR protein and *HSC82* encodes a heat shock chaperone often utilized as a control^34–37^. This was achieved using quantitative reverse transcriptase-PCR (qRT-PCR) on yeasts subjected to varying concentrations of CMX (Fig. 3c). *PGK1* was used as a housekeeping gene where all values were related to it. We found that as the concentration of CMX increases, *DFR1* expression decreases. At first, we see a possible increase in *DFR1* expression at a very mild concentration 12.5 *µ*M CMX. However, at the sub-inhibitory concentration of 37.5 *µ*M *DFR1* mRNA content was reduced by approximately 90%. No difference in the expression for *HSP82* mRNA was observed.

We then used a reporter system to further study the effect of CMX on the yeasts (Fig. 3d). If CMX affects ribonucleotide synthesis, it is likely that the pool of nucleotides will be compromised in cells treated with CMX thereby resulting in a lower rate of gene expression. Indicated by a lower activity of *β*-galactosidase, CMX was observed to reduce the level of gene expression measured by a *β*-gal reporter system (Fig. 3d). A similar effect was observed using a temperature sensitive *DFR1* knockdown strain. Shifting the cells to an inhibitory temperature of 37 °C resulted in reduced *β*-gal activity. Under this condition, the addition of CMX had no additional effect in *β*-gal expression suggesting that the effect of CMX seems to be *DFR1*-dependent.

We also examined the effect of overexpression of *DFR1* on cell sensitivity to CMX (Fig. 3e) Using a colony count assay, at the concentration of 50 *µ*M CMX control cells had significantly reduced growth compared to the cells grown without CMX. When cells were transformed with a plasmid that carries a *DFR1* gene, cell sensitivity was partially compensated. This was not true for the cells transformed with a control plasmid. As an additional control, we also examined the ability of the *DFR1* expressing plasmid to compensate for the temperature sensitive mutant for *DFR1* (Fig. 3f). At the inhibitory temperature of 37°C, colony formation was severely hindered in temperature sensitive mutants. However, introduction of the yeast *DFR1* using a plasmid returned the cell growth to the WT levels.

### CMX is predicted to interact with the DHFR catalytic domain

**Figure 4:**
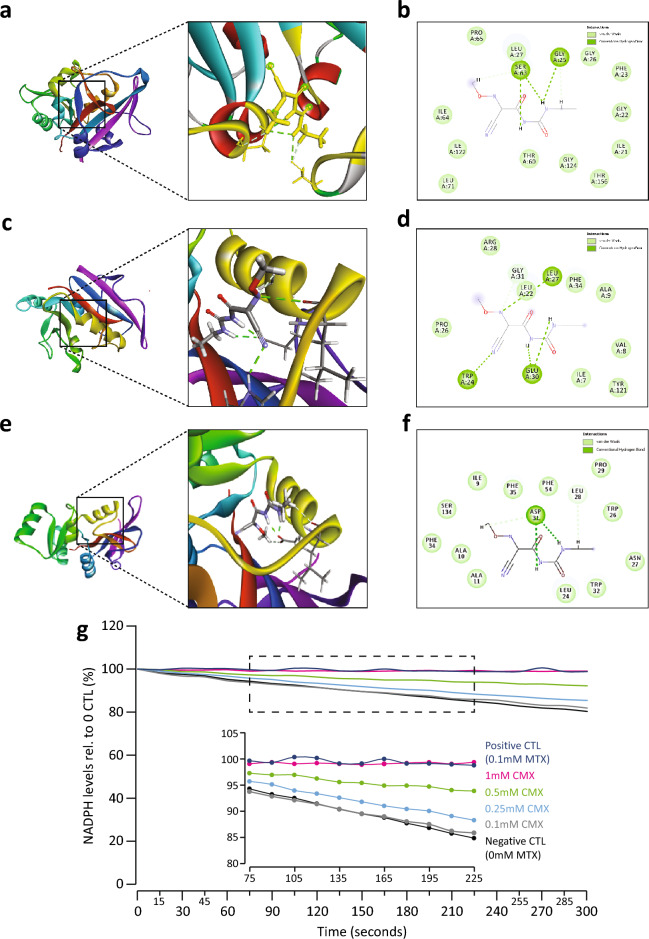
CMX inhibits the activity of mammalian DHFR. (**a**) Molecular docking simulations predict an interaction between CMX and the catalytic domain of yeast Dfr1 (∆G =  − 6.82 kcal/mol). (**b**) The residues implicated in the predicted interaction from panel (**a**). (**c**) CMX is also predicted to interact with the catalytic domain of mammalian DHFR (∆G =  − 7.17 kcal/mol). (**d**) the residues implicated in the predicted interaction from panel (**c**). (**e**) CMX is predicted to interact with the catalytic domain of the *P. infestans* DHFR protein ((∆G =  − 7.51 kcal/mol). (**f**) The residues implicated in the predicted interaction from panel (**e**). (**g**) A DHFR assay between increasing concentrations of CMX and mammalian DHFR. The presence and absence of MTX were used as experimental controls.

To further study the relationship between DHFR protein and CMX we used an in silico molecular dynamics assay. The results from the docking assay suggest that CMX is predicted to interact with the catalytic domain of the yeast DHFR (∆G = -6.82 kcal/mol, Fig. 4a). Three residues are predicted to mediate this interaction: GLY25, LEU27, and SER63 (Fig. 4b). We then assessed whether CMX is also predicted to interact with the human DHFR protein. While the human and yeast DHFR proteins share a 50% sequence similarity, the catalytic domain is highly conserved between the two as they share a 70% identity within this region. Using the same parameters, we found that CMX is also predicted to interact with the catalytic domain of human DHFR (∆G = − 7.17 kcal/mol, Fig. 4c). In addition, the predicted interaction appears to involve several residues including: LEU22, TRP24, LEU27, GLU30, and GLY31 (Fig. 4d). We then assessed whether CMX may interact with the DHFR protein of *P. infestans.* Congruent with the previous predictions, CMX is predicted to interact with the catalytic domain of *P. infestans* DHFR (∆G = − 7.51 kcal/mol, Fig. 4e). The results suggest that this predicted interaction utilizes LEU28 and ASP31 (Fig. 4f).

Next to investigate the ability of CMX to inhibit DHFR activity, we performed a standard DHFR assay between human DHFR and CMX. Methotrexate (MTX), a strong inhibitor of DHFR was used as a control. In a dose response manner, we found that DHFR activity decreases as the concentration of CMX increases (Fig. 4g). To this point, 0.5–1 mM of CMX inhibits the activity of DHFR to levels comparable to that for MTX (0.1 mM) control.

## Discussion

The data presented in this study suggests that CMX interacts with the active site of the DHFR enzyme, revealing a new possible mechanism for the compound CMX. This is supported by several lines of evidence including chemical-genomic screening data, gene expression and RNA analyses, as well as DHFR modeling and activity assays. Specifically, chemical-genomic screening resulted in two related gene families that were found to be enriched in response to CMX treatment: namely *de novo* purine biosynthesis and ribonucleoside synthesis (Figs. 1, 2d and e). This is congruent with a previous study that suggested CMX may broadly affect DNA/RNA synthesis^8^. In our screening analysis, the calcium-dependent serine protease KEX2 single-gene deletion strain displayed sensitivity to CMX (Fig. 2a–c). However, it should be noted that this gene deletion mutant has been found to be sensitive to a variety of conditions presumably due to the lack of *α*-pheromone processing^38,39^. Therefore, the observed sensitivity of the *KEX2* deletion strain to CMX may not be specific and should be interpreted with caution.

Considering the data thus far, we inferred that one of the possible targets of CMX may be the yeast DHFR enzyme Dfr1. We tested this hypothesis by first assessing the total RNA content of the cell in response to CMX and observed a dose-dependent reduction of rRNA in response to CMX. We also investigated the expression of the *DFR1* gene in response to CMX. We found that CMX affects the *DFR1* mRNA content in a dose-dependent manner (Fig. 3a–c). The *β*-gal activity assay demonstrated that loss of Dfr1 due to a shift to a restrictive temperature significantly reduces the activity of *β*-gal that are reminiscent of CMX treatment of the same strain (Fig. 3d). Addition of CMX to the cells in Dfr1 restrictive temperature had no additional effect on *β*-gal activity further connecting the activities of Dfr1 and CMX. *In-silico* molecular dynamics analysis also suggested an interaction between CMX with DHFR (Fig. 4a–f). Solidifying the prediction data, using an in vitro DHFR activity assay we found that CMX interfered with the activity of DHFR in a dose-dependent relationship (Fig. 4g).

When assessing the results from the molecular docking assay, LEU22 is predicted to be involved in the interaction between CMX and human DHFR (Fig. 4c and d) - the same residue which mediates the interaction between human DHFR and MTX^40^. Previously, clinical isolates of human DHFR substituted LEU22 with arginine thereby significantly reducing the binding affinity of MTX^40,41^, indicating that LEU22 is important for the association of MTX to DHFR. In the future, a similar approach can be applied to further investigate the potential interaction of CMX to the human DHFR. CMX was also predicted to interact with LEU27 in both tested forms of DHFR (Fig. 4a–d). Previous studies have noted the interaction between MTX and LEU27 or LEU28 in bacterial DHFR (*Lacticaseibacillus casei* and *E. coli* respectively^42^). Specifically, the *p*-aminobenzoyl group of MTX was found to interact with these residues^42^, indicating that LEU27 might be an important residue for DHFR inhibitors. MTX is also reported to interact with the conserved TRP24 and GLU30 residues in human DHFR^43^. Our predicted results for the interaction between human DHFR and CMX also utilize these residues, further supporting our prediction data (Fig. 4c and d). When assessing SER63 being predicted to be an interaction residue, previous work by Cody and colleagues noted that SER64 of the fungus *Pneumocystis carinii* DHFR (pcDHFR) forms a contact site with the pcDHFR inhibitor PY1014^43^.

We next found that CMX is predicted to interact with the *P. infestans* DHFR protein, specifically through LEU28 and ASP31 (Fig. 4e and f). Our results indicate that CMX is predicted to associate to the catalytic domain of human DHFR and can inhibit the enzymes activity in a dose-dependent manner. Inhibiting DHFR is a common anti-cancer strategy, as preventing folate metabolism results in anti-tumor proliferation. Before its widespread adoption after safety and regulatory review, CMX was postulated to be potentially carcinogenic or mutagenic to humans. Considering that CMX likely inhibits human DHFR, it is also likely that it may inhibit cellular proliferation. While further research would be required to determine physiologically relevant concentrations that would presumptively be detrimental to humans, the current agricultural application of CMX may need to undergo review considering the results presented here.

**Figure 5:**
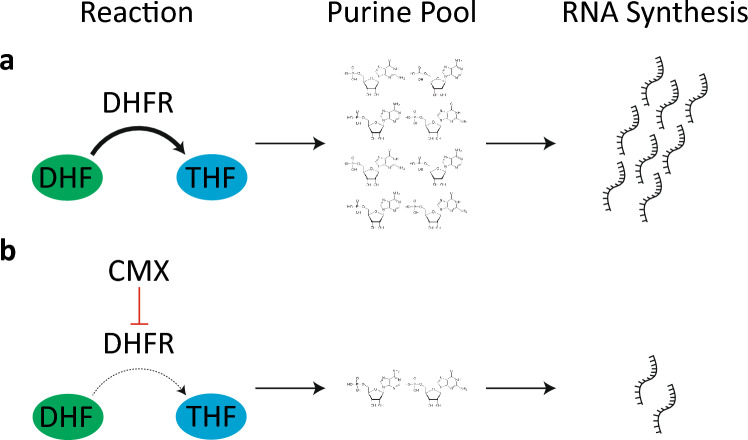
A model outlining how CMX interferes with the activity of DHFR, culminating in downstream effects and decreases in RNA synthesis. (**a**) Under normal conditions, DHFR converts dihydrofolate (DHF) to tetrahydrofolate (THF). This conversion is critical for production of purines, which are later used in RNA synthesis. (**b**) CMX inhibits the activity of DHFR, thereby reducing the conversion of DHF to THF resulting in decrease in the number of purines and therefore decreasing RNA synthesis.

In light of these observations, we propose a new model pertaining to the biocidal activity of CMX (Fig. 5). In this model, CMX interacts with the catalytic domain of the yeast DHFR enzyme Dfr1. Interaction with the catalytic domain might be mediated by several residues including LEU27. This putative interaction possibly inhibits the activity of Dfr1, thereby preventing the conversion of DHF to THF - the latter being an important cofactor in purine biosynthesis and cell metabolism^44^. Consequentially, RNA biosynthesis is inhibited as purines are no longer synthesized. This culminates in several off-target effects including potentially affecting RNA biosynthesis. The data presented in this study therefore offer possible targets for future studies to dissect the proposed interaction between CMX and DHFR. Taken together, our results suggest that CMX disrupts RNA biosynthesis through a probable association with the active site of yeast and human DHFR.

## Methods

### Strains and reagents

Deletion strains in BY4741 (*MATa orf*∆*: kanMX4 his3*∆*1 leu2*∆*0 met15*∆*0 ura3*∆*0*) were obtained from the yeast single-gene deletion collection, originally purchased from ThermoFisher Canada^45^. Single-gene deletion strains were verified using PCR analysis. All reagents were purchased from ThermoFisher Canada unless otherwise stated. Primers for qRT-PCR were obtained from Integrated DNA Technologies (IDT) Canada.

### Chemical-genomic screen

To perform the chemical-genomic screen, we first subjected a random collection of 384 single-gene deletions (1 plate of the single-gene deletion set) to varying concentrations of CMX. Once a suitable sub-inhibitory concentration was determined, we then performed the large-scale screening using the entire deletion set in the presence of 75 *µ*M CMX in duplicate. Any yeasts that displayed sensitivity were subsequently transferred onto a new single plate. We then repeated the screening to verify the gene-deletion candidates from the large-scale screening in biological triplicate. The plates were photographed, and the pictures were uploaded into Growth Detector (GD)^32^. Colony size was measured in GD by assessing the pixels corresponding to the colonies. These pixels were measured and normalized over the average growth of the colonies on that plate and related to that of the control plate as described in Ref.^32^. Reductions in colony growth are reported as a heat map across the 49 strains. The verified candidates were then subjected to drug sensitivity and colony-counting analysis.

For drug sensitivity analysis, yeasts were grown from independent colonies for 17 hours at 30 °C in liquid YPD. Spot test analysis of serial dilutions of cell suspensions were spotted onto solid media in the presence or absence of CMX. For growth sensitivity to CMX, 75 *µ*M was used in media as described previously^46^. Sensitivity to the compound was assessed by comparing the number and size of the colonies formed on each plate after 48 hours in comparison with wild type. To measure colony size, the corresponding plates were photographed and uploaded into Fiji/ImageJ software. The pictures were converted to grey-scale and colony size was measured using a circular region of interest (outlining the circumference of each yeast spot) using pixel white/black intensity. The resulting values were then normalized to the WT of each plate.

To perform the colony counting assay, yeasts from independent colonies were grown for 17 hours at 30 °C in liquid YPD. 100 *µ*L of diluted (10^*−*3^) cell cultures were subsequently transferred onto YPD plates in the absence and presence of CMX. The colonies were counted two days after incubation at 30 °C. Each experiment was repeated at least three times. A *t*-test analysis (*p* ≤ 0*.*05) was used to determine statistically significant differences. The remaining candidates were then subjected to Gene Ontology (GO) analysis to determine enrichment and significance.

### Quantitative realtime-PCR (qRT-PCR)

The content of mRNAs was evaluated using qRT-PCR analysis. Deletion mutants in BY4741 were grown in YPD overnight with or without CMX treatment. Total RNA was extracted with Qiagen^®^ RNeasy Mini Kit as described previously^47^. Complementary DNA (cDNA) was synthesized using iScript Select cDNA Synthesis Kit (Bio-Rad^®^) according to the manufacturer’s instructions. cDNA was then used as a template for quantitative PCR. qRT-PCR was carried out using Bio-Rad^®^ iQ SYBR Green Supermix and the CFX connect real-time system (Bio-Rad^®^), according to the manufacturer’s instructions. *PGK1* was used as a constitutive reference gene (internal control). The procedure and data analysis were performed according to MIQE guidelines^48^. In brief, total RNA extraction was performed in biological triplicate, with the quality and quantity of RNA assessed via spectrophotometry and gel electrophoresis. Approximately 1 µg of RNA was used to synthesize cDNA. CT values were normalized to the *PGK1* internal control through subtraction with the subsequent values averaged and normalized to the control for *HSP82* or *DFR1* respectively. These values were then transformed to *ΔΔCT* scores. The primers used were designed to 60 nucleotides prior to the sequence in the flanking region of the indicated gene. *PGK1* Forward: CAGACCATTCTTGGCCATCT; *PGK1* Reverse: CGAAGATGGAGTCACCGATT; *HSC82* Forward: CTTGTTTTCTTTTTCTTGAAACGCTAC; *HSC82* Reverse: GTCAATCGTAAGTGTACACTAAACTTT *DFR1* Forward: AGTTAACATTATGCTTTGCATGATAAT; *DFR1* Reverse: GAGGCTTATCAGTTCTATCACTATTTA. *PGK1* primer sequences were designed previously described^49^. Significance was determined with a t-test analysis (*p* ≤ 0*.*05). Error bars are derived using a standard deviation (3 replicates).

### Quantitative β-galactosidase assay

The effect of CMX on translation in different yeast strains were examined using LacZ reporter systems. To evaluate the activity of LacZ expression cassettes, quantitative *β*-galactosidase assay was performed using ONPG (O-nitrophenyl-*α*-d-galactopyranoside) and the p416GAL1-LacZ plasmid as previously described^50,51^. LacZ expression was induced by incubating yeasts in YP-Galactose for 6 hours at 37 °C. Each experiment was repeated at least three times. T-test analysis (*p* ≤ 0*.*05) was used to determine statistically significant differences.

### Molecular docking

The crystal structure and homologues of the DHFR proteins with appropriate domains were obtained from the Protein Data Bank database using the protein-specific local alignment search method utilizing Position-Specific Iterated BLAST. The homology model of the DHFR domains was created using homologs with the highest sequence similarity using the Swiss Model, a fully automated platform for modeling protein structure homology. Using 3DRefine and Phyre2, protein refinement and homology sequencing studies are conducted to develop credible protein models for detecting the likelihood of binding site predictions. The 3D structure of CMX is generated and docked to the protein structure for docking predictions using PyMol and the High Ambiguity Driven Protein-protein Docking tool. NAMD version 2.11b was used for molecular dynamics simulations, which utilizes an Amber force field including substrate peptide and additional enzyme cofactors. To exclude any overlaps in the simulation data, the interference ratio contains a minimum of 10,000 steps with a time stage of 2 fs and a temperature of 298 K using the Generalized Born Implicit Solvent. Path data were examined using PyMol and extra plug-ins to determine further hydrogen bonds, RMSD, RMSF, salt bridges, and other interacting bond forms during the DHFR-CMX interaction.

### DHFR activity assay

DHFR activity in the presence of CMX was assessed using the Dihydrofolate Reductase assay kit from Sigma-Aldrich Canada according to the manufacturer’s instructions. Briefly, 1.5E–03 units of DHFR were subjected to increasing concentrations of CMX. The DHFR enzyme was incubated with the CMX for a total of 5 minutes per concentration. DHFR in the presence and absence of 0.1 mM MTX were used as controls. The assay was repeated in technical triplicate with *T*-test analysis (*p* ≤ 0*.*05) used to determine significance.

### Supplementary Information

Supplementary Figure 1.

## Data Availability

The datasets generated during and/or analyzed during the current study are available from the corresponding author on reasonable request.
