# Supplementary figures and images for: Cymoxanil disrupts RNA synthesis through inhibiting the activity of dihydrofolate reductase
Source: Sci Rep. 2024 May 22;14:11695. doi: 10.1038/s41598-024-62563-5 (PMC11111663; doi:10.1038/s41598-024-62563-5)

Total RNA

25S  
18S

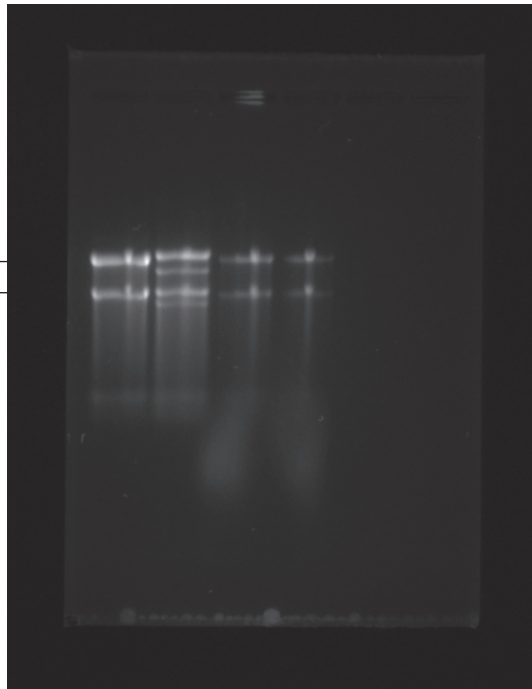

0.0 12.5 25.0 37.5  
Cymoxanil ( $\mu\text{M}$ )

Supplement: Supplementary file 1 — Supplementary Figure 1. [file 41598_2024_62563_MOESM1_ESM.pdf]
